# 33 Ironman triathlons in 33 days–a case study

**DOI:** 10.1186/2193-1801-3-269

**Published:** 2014-05-28

**Authors:** Beat Knechtle, Christoph Alexander Rüst, Thomas Rosemann, Normand Martin

**Affiliations:** Facharzt FMH für Allgemeinmedizin, Gesundheitszentrum St. Gallen, Vadianstrasse 26, 9001 St. Gallen, Switzerland; Institute of General Practice and for Health Services Research, University of Zurich, Zurich, Switzerland; Centre de Médecine Sportive de Laval, Laval, Québec Canada

**Keywords:** Swimming, Cycling, Running, Multi-sport, Ultra-endurance

## Abstract

This case report presents the performance of an athlete who completed for the first time in history the total distance of 33 Ironman triathlons within 33 consecutive days. The athlete finished the total distance of 7,458 km (*i.e.* 125 km swimming, 5,940 km cycling and 1,393 km running) within a total time of 410 h and a mean time of 12 h 27 min per Ironman distance. During the 33 days, the athlete became slower in swimming (r^2^ = 0.27, *p* = 0.0019), transition time 1 (r^2^ = 0.66, *p* < 0.001), and transition time 2 (r^2^ = 0.48, *p* < 0.0001). However, in cycling (r^2^ = 0.07, *p* = 0.13), running (r^2^ = 0.04, *p* = 0.25) and overall race time (r^2^ = 0.10, *p* = 0.069), the athlete was able to maintain his performance during the 33 days. The coefficients of variation (CV) for the split times in swimming, cycling, running and overall race times were very low (*i.e.* 2.7%, 3.2%, 4.7%, and 2.7%, respectively) whereas the CV for transition times 1 and 2 were considerably higher (*i.e.* 25.5% and 55.5%, respectively). During the 33 days, body mass decreased from 83.0 kg to 80.5 kg (r^2^ = 0.55, *p* < 0.0001). Plasma [Na^+^] remained within the reference range, creatine kinase, blood glucose and liver enzymes were minimally elevated above the reference range after four of five stages where blood analyses were performed. This case report shows that this athlete finished 33 Ironman triathlons within 33 consecutive days with minor variations over time (*i.e.* even pacing) in both split times and overall race times. This performance was most probably due to the high experience of the athlete, his pacing strategy and the stable environmental conditions.

## Background

An ultra-endurance performance is defined as any endurance performance of six hours in duration or longer (Zaryski and Smith 2005). In multi-sports disciplines such as triathlon, an Ironman triathlon covering 3.8 km swimming, 180 km cycling and 42.2 km running with the fastest winner times of ~8 hours has to be considered as an ultra-endurance performance (Lepers 2008). Apart from the classical Ironman distance, triathlon races with multiple Ironman distances do exist such as the Double Iron ultra-triathlon (*i.e.* 7.6 km swimming, 360 km cycling and 84.4 km running), the Triple Iron ultra-triathlon (*i.e.* 11.4 km swimming, 540 km cycling and 126.6 km running), the Deca Iron ultra-triathlon (*i.e.* 38 km swimming, 1,800 km cycling and 422 km running) and the Double Deca Iron ultra-triathlon (*i.e.* 76 km swimming, 3,600 km cycling and 844 km running) (Knechtle et al. 2011; Lenherr et al. 2012; Lepers et al. 2011; Rüst et al. 2014).

In addition to the classical Ironman triathlon held as a single stage race, also multi-stage races with consecutive Ironman triathlons held for several days are known (Herbst et al. 2011; Knechtle et al. 2012a). Actually, the Deca Iron ultra-triathlon covering ten Ironman triathlons within ten days is the most popular multi-stage race (Herbst et al. 2011; Knechtle et al. 2008a, [b], 2012a). Recent studies showed that performance for each Ironman triathlon during a Deca Iron ultra-triathlon decreased with increasing duration of the race (Knechtle et al. 2012a) where the fastest Ironman was achieved on the first day (Day 1) (Herbst et al. 2011; Knechtle et al. 2008a) and the slowest on the last day (Day 10) (Herbst et al. 2011).

A Deca Iron ultra-triathlon is a highly selective race and less than 50% of the starters are able to finish (Herbst et al. 2011; Knechtle et al. 2012a). For the very long ultra-triathlon distances (*i.e.* Deca Iron ultra-triathlon), previous experience seems of outmost importance (Lepers et al. 2011). For a successful finish in a Deca Iron ultra-triathlon, the most important predictor variables were extensive previous experience since both the number of finished Triple Iron triathlons and the personal best time in a Triple Iron triathlon were related to overall race time (Herbst et al. 2011).

Since the first edition of a Deca Iron ultra-triathlon in 2006 in Monterrey, Mexico (Knechtle et al. 2008a, 2011), several races of this kind have been held (Herbst et al. 2011; Knechtle et al. 2008a, [b], 2012a). To date, the edge of human limits in ultra-triathlon was the Double Deca Iron ultra-triathlon covering the total distance of 20 Ironman triathlon races (Lenherr et al. 2012; Rüst et al. 2014). However, in 2013, one ultra-triathlete completed for the first time in history the total distance of 33 Ironman distances within 33 consecutive days (http://pse33.ca/intro/).

Little is known about the pacing strategy in endurance and ultra-endurance performance (Abbiss and Laursen 2008; Herbst et al. 2011). Actual evidence suggests that well-trained athletes tend to adopt a positive pacing strategy during endurance and ultra-endurance performance, whereby the athlete progressively slows after peak speed is reached (Abbiss and Laursen 2008). The underlying mechanisms influencing the regulation of pace during exercise are unclear (Abbiss and Laursen 2008). It has been suggested, however, that self-selected exercise intensity is regulated within the brain based on a complex algorithm involving peripheral sensory feedback and the anticipated workload remaining (Abbiss and Laursen 2008). In a Deca Iron ultra-triathlon, the continuous loss in body mass (Herbst et al. 2011) and the growing energy deficit during the race (Knechtle et al. 2008b) might limit the performance of the athletes.

To date, to the best of our knowledge, finishing the total distance of 33 Ironman triathlons within 33 days represents the longest distance ever completed by a triathlete. In this case report, we report the changes in performance in split and overall race times, in body mass, and in selected laboratory measurements. Based upon previous reports for Deca Iron ultra-triathlon, it was hypothesized that performance in both split times and overall race times would decrease over days.

## Case description

### Ethics

This study was approved by the Institutional Review Board of St. Gallen, Switzerland, with a waiver of the requirement for informed consent given that the study involved the analysis of publicly available data. The data set for this study was obtained from the website of the athlete http://pse33.ca/33-ironman-resultats-quotidiens/. Additional informed consent was obtained from all participants for whom identifying information is included in this article. The athlete agrees to the analysis and publication of his data as presented in this article.

### The event

From June 25, 2013, to July 27, 2013, the ultra-triathlete performed daily one Ironman distance triathlon (*i.e.* 3.8 swimming, 180 km cycling and 42 km running) for 33 consecutive days in Laval, Québec, Canada. The Ironman triathlons were held each day on the same course. Prior to the start of the event, it was the intention of the athlete to complete the total number of 33 Ironman triathlons and to finish each day the Ironman distance within 12 hours.

Swimming was held in a heated 18 m indoor-pool with a mean temperature of 29°C. During swimming, the athlete was wearing a wetsuit ‘Nineteen Rouge’ (http://www.nineteenwetsuits.com). Cycling was held on a lap of 1,652 m open to public traffic. During the cycling split, he was riding a ‘Specialized Tarmac Ultegra 2013’ (http://www.specialized.com/ch/de/bikes/road/tarmac) as a conventional road bike without a speedometer, nor a cadence monitor, nor a power tap. Running was held on a lap of 2 km. Environmental conditions are presented in Table 1 with temperatures and precipitations.

**Table 1:** **Environmental conditions during the event**

Day	Maximum temperature (°C)	Minimum temperature (°C)	Mean temperature (°C)	Precipitation during the day (mm)
**1**	27.8	20.5	27.6	4.2
**2**	31.0	19.9	25.4	
**3**	24.5	18.6	21.6	
**4**	22.5	17.3	19.9	16.2
**5**	24.1	16.5	20.3	
**6**	25.6	15.6	20.6	
**7**	23.8	16.3	20.1	
**8**	22.9	17.0	19.9	
**9**	26.5	17.8	22.1	1.2
**10**	28.3	20.5	24.4	3.2
**11**	25.4	19.7	22.6	28.6
**12**	29.0	19.1	24.1	0.4
**13**	27.8	20.6	24.2	26.6
**14**	29.1	19.6	24.4	126
**15**	25.4	19.9	22.6	0.6
**16**	30.6	17.7	24.1	0.6
**17**	25.4	15.7	20.6	
**18**	25.2	15.2	20.2	
**19**	27.4	16.0	21.7	
**20**	29.7	19.2	24.4	
**21**	31.2	22.0	26.6	
**22**	32.7	22.1	27.4	
**23**	34.8	24.1	29.4	
**24**	33.7	23.7	28.7	
**25**	34.9	20.9	27.9	8.6
**26**	28.6	19.2	23.9	1.6
**27**	23.0	15.6	19.3	
**28**	23.9	16.4	20.1	
**29**	28.8	15.9	22.4	
**30**	21.5	13.1	17.3	
**31**	23.3	12.6	17.9	
**32**	24.0	13.5	18.8	
**33**	23.0	18.1	2.06	10.2
**mean**	27.1	18.1	22.1	6.9

Data are retrieved from http://www.meteomedia.com/meteo/historical-weather/canada/quebec/laval/.

During swimming, cycling and running, he was followed and supported by a large support crew of ~30 persons. Three times per day (*i.e.* before the swimming, at noon during cycling and after the finish of a stage), the family physician of the athlete passed to look for eventual problems. Each morning before the start of a stage, body mass was measured using a conventional balance to the nearest 0.5 kg (http://www.danze.com).

Food and fluid intake was not recorded in details. Before and after a stage, the athlete was eating at his home. During a stage, food and fluids were provided by a large support crew of more than 30 persons during the 33 stages. In the morning at home, the triathlete consumed a conventional continental breakfast with bread, butter, jam, honey and eggs. He additionally consumed 30 g of a protein powder in a milk drink. During swimming, he consumed water and pieces of bananas. During cycling, he was provided with solid food such as meat, fish, pasta, mashed potatoes, and fruits. He consumed no energy bars or carbohydrate gels. As fluids, he consumed water, protein drinks and fruit juices. During running, he was provided the same food and fluids as during cycling, however, the amounts were smaller. In the evening at home, he consumed conventional meals rich in carbohydrates and protein.

### Blood sampling and blood analyses

The family physician of the athlete took a first blood sample 11 days before the event. During the event, blood samples were taken on Day 7, Day 13, Day 16, Day 19, Day 24 and Day 29. After the event, blood samples were drawn 7 days and 78 days after the finish. All samples were drawn after the stages at home of the athlete and analysed in the laboratory of Hôpital du Sacré-Coeur de Montréal, Québec, Canada.

### Data analyses

Data in the text are given as mean ± standard deviation (SD). Split times and overall race times of each stage were converted to minutes. The coefficient of variation (CV) of performance (CV % = 100 × SD/mean) was calculated for split times and overall race times. A potential change in a variable over time (*i.e.* split times, transition times, and overall race times) was investigated using linear regression analyses. Pearson’s correlation coefficients were used to assess associations between various variables. Statistical analyses were performed using Statsoft, Version 6.1, Statistica, Tulsa, OK, USA. Significance was accepted at *p* < 0.05 (two-sided for *t*-tests).

### Results

The athlete completed the total distance of 7,458 km (*i.e.* 125 km swimming, 5,940 km cycling and 1,393 km running) within a total time of 410 h. The mean of the split times for swimming, cycling and running, the transition times and the overall race times including the CV are presented in Table 2.

**Table 2:** **Mean (±SD) for split times, transition times (T1 and T2) and overall race times for 33 Ironman distance triathlons**

	3.8 km swimming	T1	180 km cycling	T2	42 km running	Overall race time
Mean (min)	58.7	11.6	371.1	6.7	296.4	747.5
SD (min)	1.6	2.9	12.0	3.7	14.1	20.8
CV (%)	2.7	25.5	3.2	55.5	4.7	2.7

T1 = transition time 1 from swimming to cycling; T2 = transition time 2 from cycling to running.

**Figure 1:**
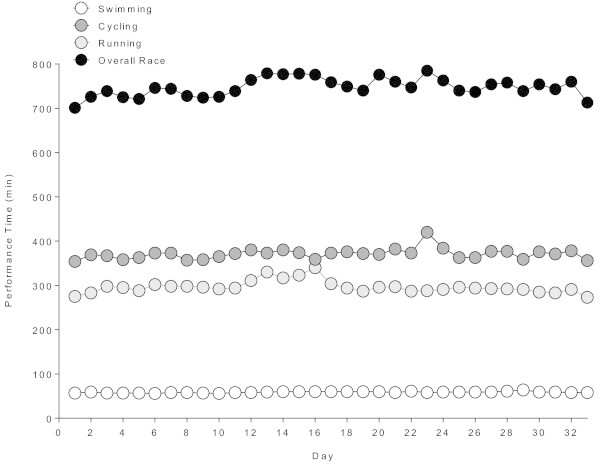
**Changes in split times and overall race times across the 33 days.**

**Figure 2:**
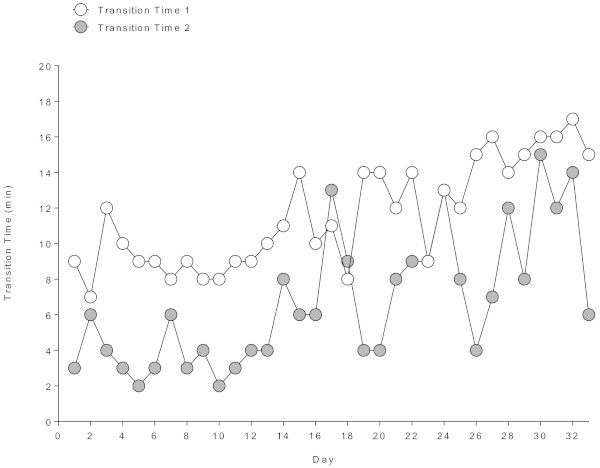
**Changes in transition times across the 33 days.**

The changes in split times and overall race times across the 33 days are shown in Figure 1, the changes in transition times in Figure 2. The athlete became slower in swimming (r^2^ = 0.27, *p* = 0.0019), transition time 1 (r^2^ = 0.66, *p* < 0.001), and transition time 2 (r^2^ = 0.48, *p* < 0.0001). In cycling (r^2^ = 0.07, *p* = 0.13), running (r^2^ = 0.04, *p* = 0.25) and overall race time (r^2^ = 0.10, *p* = 0.069), however, the athlete was able to maintain his performance during the 33 days.

His split times on Day 1 for swimming, cycling, running and overall race time were 57 min 37 s, 5 h 54 min 57 s, 4 h 35 min 43 s, and 11 h 41 min 17 s, respectively. For the last day, the times were 58 min 20 s, 5 h 56 min 26 s, 4 h 33 min 28 s, and 11 h 53 min 22 s, respectively.

The analysis of the blood samples (Table 3) showed that haemoglobin before, during and after the event was below the reference range, blood glucose was above the reference range after some stages, liver enzymes and creatine kinase were minimally above the reference range during the event, and both total and low-density lipoprotein cholesterol were below the reference range before and after the event.

**Table 3:** **Results of the laboratory analyses**

Parameter and normal range	- 11 d	D 7	D 13	D 16	D 21	D 24	D 29	+ 3 d	+ 73 d
White blood cells (4.0–11.0 × 10^9^/l)	4.1	5.4	6.4	5.5	5.1	6.2	5.3	4.2	4.5
Red blood cells (4.50–5.90 × 10^12^/l)	4.15	4.39	4.35	4.41	4.37	4.54	4.47	4.50	4.44
Haemoglobin (140–175)	129*	138*	134*	137*	138*	142	143	139*	139*
Sodium (135–150 mmol/l)	143	142	143	140	138	143	140	139	139
Potassium (3.5–5.3 mmol/l)	4.3	4.7	4.3	4.7	4.8	4.5	4.6	4.8	4.5
Glucose (3.9–6.1 mmol/l)	4.7		4.8	8.3*		5.6	6.5	7.2*	4.7*
Creatinine (53–120 mmol/l)		65	70	66	75	62	65		78
Calcium (2.02–2.65 mmol/l)		2.21	2.15	2.19	2.19	2.27	2.24	2.25	2.29
Phosphate (0.80–145 mmol/l)		1.10	1.08	0.87	0.94	1.16	1.05	0.81	1.42
Magnesium (0.70–1.05 mmol/l)		0.94	0.96	0.88	0.85	0.93	0.94	0.85	0.94
AST (0–40 U/l)			38	47*	48*	46*	46*	33	21
ALT (0–40 U/l)		39	39	46*	49*	50*	58*	47*	15
ALP (40–120 U/l)			44	46	46	46	48	49	39
Amylase (0–100 U/l)		40	57	40	72	39	39	40	30
Lipase (7–60 U/l)			78	49	101*	41	42	30	22
Total protein (62–84 g/l)	65		67	70	70	71	71	71	67
Albumin (35–50 g/l)	41			43	44	44	44	45	43
γ-GT (8–61 U/l)									12
Cholesterol (4.20–5.20 mmol/l)	3.69*							5.19	3.70*
Triglycerides (0.6–2.30 mmol/l)	0.73							0.95	0.67
LDL-cholesterol (2.20–3.40 mmol/l)	1.60*							3.01	1.77*
HDL-cholesterol (0.90–1.80 mmol/l)	1.75							1.72	1.62
VLDL-cholesterol (0.26–1.03 mmol/l)	0.34							0.44	0.31
TSH (0.5–4.80 mU/l)	1.63								0.98
Iron (11–28 μmol/l)	14							23	16
UIBC (20–66 μmol/l)	32							29	30
TIBC (52–77 μmol/l)	46							52	46
Transferrin (1.70–3.40 μmol/l)	1.79							2.03	1.79
Transferrin saturation (15–40%)	23							34	27
Ferritin (25–250 μg/l)	72.3								66.4
CK (12–200 U/l)		469*	391*		447*		311*		
INR (0.80–1.50)								0.93	
PTT (28.0–42.0)								34.8	
Fibrinogen (1.50–4.50)								3.36	
Vitamin B_12_ (138–781 pmol/l)	289							595	
Folic acid (> 58 pmol/l)	> 58							> 58	
CRP (‹ 5.0)	0.4								

AST = aspartate aminotransferase, ALT = alanine aminotransferase, ALP = alkaline phosphatase, γ-GT = gamma glutamyltransferase, LDL = low-density lipoprotein, HDL = high-density lipoprotein, VLDL = very low density lipoprotein, TSH = thyroid-stimulating hormone, UIBC = unsaturated iron binding capacity, TIBC = total iron binding capacity, CK = creatine kinase, INR = international normalized ratio, PTT = partial thromboplastin time, CRP = C-reactive protein. * = out of reference range.

**Figure 3:**
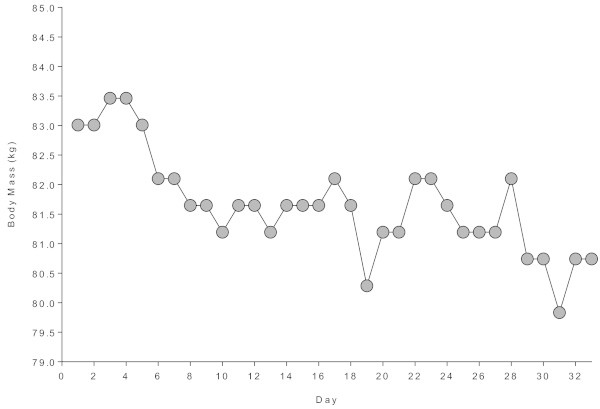
**Changes in body mass across the 33 days.**

The athlete suffered during the event from some minor problems of the toes such as blisters, a subungual hematoma, a hypoglycaemia on Day 10, a synovitis of the left ankle on Day 11 and Day 17, a tendinitis of the long extensor of the first toe of the left foot on Day 12 and 19, and a femoral-patellar syndrome of both knees starting from Day 20 until the end. His body mass decreased across the 33 days from 83.0 to 80.5 kg (r^2^ = 0.55, *p* < 0.0001) (Figure 3).

## Discussion

The intention of this case report was to report the changes in performance in split times, transition times, overall race times, body mass and selected laboratory values in an event covering 33 consecutive Ironman triathlons. Based upon previous reports for Deca Iron ultra-triathletes it was hypothesized that the performance in both split times and overall race times would decrease over days. Indeed, swimming split times increased minimally during the 33 days whereas, however, split times in cycling and running and overall race times remained unchanged.

The difference between the first and the last Ironman for swimming, cycling, running and overall race time was 43 s, 2 min, 2 min, and 12 min, respectively. Expressed in percent, the differences were 1.24%, 0.56%, 0.72%, and 1.56%, respectively. Swimming, cycling and overall race time was slower on Day 33, but running was faster on Day 33. Comparing the percent changes with athletes competing in a Deca Iron ultra-triathlon (Herbst et al. 2011), the changes were 18.5 ± 19.5%, 27.0 ± 29.0%, 22.5 ± 32.0%, and 24.0 ± 21.0%, respectively, where athletes became slower in both the split disciplines and overall race times. An interesting finding was that the CV for split times in swimming, cycling, running and overall race times were very low (*i.e.* 2.7%, 3.2%, 4.7%, and 2.7%, respectively) whereas the CV for transition times 1 and 2 were considerably higher (*i.e.* 25.5% and 55.5%, respectively). Most probably the athlete focussed more on the performance in the split disciplines than on the transition times. Otherwise, he may have used the transition times for eating and drinking. However, the aspect of transition times in Ironman triathlons needs further investigation. Reducing the transition times might improve overall race time.

### The aspect of pacing strategy

The athlete stated prior to the start of the event to finish the total number of 33 Ironman triathlons and intended to finish each Ironman within 12 hours in his self-paced event. Pacing in endurance is very complex (Abbiss and Laursen 2008). Pacing strategies are the consequence of a complex regulation and serve a dual role: they are both the result of homeostatic regulation by the brain, as well as being the means by which such regulation is achieved (Atkinson et al. 2007). The ‘algorithm’ of pacing strategy is sited in the brain and needs afferent input from ‘interoceptors’ (*e.g.* as heart rate and respiratory rate), as well as ‘exteroceptors’ (*e.g.* local environmental conditions (Atkinson et al. 2007).

A potential explanation for the even pacing (*i.e.* unchanged split times in cycling and running and unchanged overall race times) could be the environmental conditions (*i.e.* unchanged course for 33 days) and the stable weather (*i.e.* minimal rain). Abbiss and Laursen ( 2008) reported that a constant pace is ‘optimal’ for prolonged locomotive events such as running, swimming, rowing, skiing, speed skating and cycling under stable external (*i.e.* environmental and geographic) conditions. A further explanation could be the unchanged course (*i.e.* every day the same course) during the 33 days where the athlete got very familiar with over time. An even distribution of power output (*i.e.* even pacing) is both physiologically and biophysically optimal for longer (*i.e.* > 4 km) time-trials held in conditions of unvarying wind and gradient (Atkinson et al. 2003).

Prior to the start of the event, the athlete intended to finish each Ironman consistently within 12 hours. This intention might have been the key for the regular pace since the process of pacing has been associated with the goal-directed regulation of exercise intensity across an exercise bout (Smits et al. 2014). The selection and distribution of work rate is one of the many factors influencing cycling speed (Atkinson et al. 2007). Also the intention of a selection of a certain speed is of importance. Decisions relating to the setting of an appropriate goal and the overall strategic approach to be used are made prior to the start of an event whereas tactical decisions are made during the event (Renfree et al. 2014). The self-selected pace is of importance for the outcome of the race. Renfree et al. ( 2012) showed that time trials cyclists with a more aggressive strategy (*i.e.* with a fast start) had higher levels of positivity than cyclists who started slower in the time trial.

### The aspect of previous experience

Since personal best time in an Olympic distance triathlon was a predictor variable in Ironman triathlon (Rüst et al. 2011) and both the number and the personal best time in a Triple Iron ultra-triathlon were both highly predictive for the performance in a Deca Iron ultra-triathlon (Herbst et al. 2011), we summarized the number of completed Triple Iron ultra-triathlons with the personal best time with data available from the website of the athlete and his personal records (Table 4). In addition to the data of Triple Iron ultra-triathlon, we additionally inserted data from single Ironman, Double Iron ultra-triathlon and longer ultra-triathlons since personal best time in shorter events are highly predictive for performance in longer events (Herbst et al. 2011; Rüst et al. 2011). Considering previous findings, the athlete has a personal best time in Triple Iron ultra-triathlon of 2,199 min and the athletes in the study of Herbst et al. ( 2011) of 2,577 min. Therefore, the personal best time of our athlete is 378 min (*i.e.* 14.7%) faster than the personal best time of the athletes in the study of Herbst et al. ( 2011). The greater experience of the present athlete is most probably the reason for the minimal variation in split times and overall race times across the 33 days. Renfree and St Clair Gibson ( 2013) investigated pacing strategies in female marathoners regarding their performance level and personal best time. The fastest runners achieved faster races times in relation to their personal best marathon time than athletes in other groups, who selected unsustainable initial speeds resulting in subsequent significant losses of running speed. The authors suggested that psychological factors specific to a major competitive event influenced decision making. However, the pre-race ‘history’ may be limited to predict a successful race outcome. Marongiu et al. ( 2013) showed that pre-race tests are of limited use to predict race outcome in Ironman triathletes.

**Table 4:** **Number of finished triathlons with personal best time and the year (in parentheses) when the personal best time was achieved**

Race distance	Number of finished races	Personal best time (h:min)
Olympic distance triathlon	many	2:05
Ironman	many	10:25 (2005)
Double Iron ultra-triathlon	11	21:48 (2005)
Triple Iron ultra-triathlon	6	36:39 (1998)
Deca Iron ultra-triathlon	1	297:42 (2002)

### Changes in laboratory values and body mass

The analysis of the blood-related parameters and body mass showed some minor changes across the 33 days and in the post-race period. The athlete lost ~2 kg of body mass during the 33 days. This is comparable to the 1.8 ± 2.1 kg loss in body mass of the 23 Deca Iron ultra-triathletes described by Herbst et al. ( 2011). A similar result has been reported by Knechtle et al. ( 2012a) with a loss of 1.7 ± 2.4 kg body mass in 11 finishers in a Deca Iron ultra-triathlon. In contrast to the Deca Iron ultra-triathletes described in Herbst et al. ( 2011) and Knechtle et al. ( 2012a) where performance decreased with increasing race duration, the present athlete was able to maintain his performance across the 33 days. A potential explanation could be the decrease in body mass as an ‘ergogenic effect’. For example, for ultra-runners competing in a 100-km ultra-marathon, faster runners lost more body mass than slower runners (Knechtle et al. 2012b). Also in multi-stage ultra-marathoners competing in the ‘Marathon des Sables’, a significant loss in body mass did not systematically affect race performance (Zouhal et al. 2009). Similarly, in marathoners, a loss in body mass was inversely related to race time (Zouhal et al. 2011). The loss in body mass was > 3% in runners completing the marathon in less than 3 hours (Zouhal et al. 2011).

In Ironman triathletes, the decrease in body mass is due to a decrease in both fat and lean mass where the loss in lean mass was explained by a decrease in muscle density as an indicator of glycogen loss (Mueller et al. 2013). The loss in glycogen might be replenished during the night in a multi-stage triathlon (Cermak and van Loon 2013). Athletes in a Deca Iron ultra-triathlon lost ~3 kg of body fat whereas skeletal muscle mass, mineral mass and body water were unchanged after the race (Knechtle et al. 2008a). The continuous decrease in body fat during the race, however, might be of importance to improve race performance since body fat showed a relationship to total race time in male Ironman triathletes (Knechtle et al. 2010). The lower the body fat, the faster the Ironman race time (Knechtle et al. 2010).

During the event, liver enzymes and creatine kinase showed a minimal increase above the reference range. An increase in these parameters is a common finding in ultra-endurance athletes such as ultra-marathoners (Kłapcińska et al. 2013; Waśkiewicz et al. 2012; Wu et al. 2004) and does not present a serious health risk (Kłapcińska et al. 2013). An interesting finding was that plasma sodium concentration [Na^+^] was constantly within the reference range and no situation of exercise-associated hyponatremia (EAH) occurred. In Ironman triathletes, the prevalence of EAH can reach ~18% (Speedy et al. 1999) and triathletes competing in distances longer than the Ironman seemed to be at a higher risk to develop EAH (Rüst et al. 2012). Overall, only minimal changes were detected in the laboratory analyses while the athlete was asymptomatic during the whole event.

### Limitations

This case report was not started with the intention to perform a scientific study to write a case report. The first intention of the athlete and his support crew was to achieve the 33 Ironman distances within 33 days. The idea was to secure a perimeter near facilities since there was no funding for the event and it was impossible to have a racing caravan. Unfortunately, sleep and eating pattern were not recorded. Too many people were involved in feeding and supporting the athlete and it was impossible to record accurately the ingested calories and the timing of feeding.

## Conclusion

This case report shows that this athlete finished 33 Ironman triathlons within 33 consecutive days with minor variations (*i.e.* even pacing) in both split times and overall race times over time. This performance was most probably due to the high experience of the athlete, his pacing strategy and the stable environmental conditions. Minimal changes were detected in the laboratory analyses and body mass while the athlete was asymptomatic during the whole event.
